# Chromosome band 11q23 deletion predicts poor prognosis in bone marrow metastatic neuroblastoma patients without *MYCN* amplification

**DOI:** 10.1186/s40880-019-0409-1

**Published:** 2019-11-04

**Authors:** Zhi-Xia Yue, Tian-Yu Xing, Chao Gao, Shu-Guang Liu, Wen Zhao, Qian Zhao, Xi-Si Wang, Mei Jin, Xiao-Li Ma

**Affiliations:** Beijing Key Laboratory of Pediatric Hematology Oncology, National Key Discipline of Pediatrics, Ministry of Education, MOE Key Laboratory of Major Diseases in Children Hematology Oncology Center, Beijing Children’s Hospital, Capital Medical University, National Center for Children’s Health, 56 Nanlishi Road, Beijing, 100045 People’s Republic of China

**Keywords:** Neuroblastoma, *MYCN* amplification, 11q23 deletion, Fluorescence in situ hybridization, Bone marrow metastasis, Event-free survival, Overall survival, Neuron-specific enolase, Lactate dehydrogenase

## Abstract

**Background:**

Interphase fluorescence in situ hybridization (FISH) of bone marrow cells has been confirmed to be a direct and valid method to assess the v-myc avian myelocytomatosis viral oncogene neuroblastoma derived homolog (*MYCN*) amplification in patients with bone marrow metastatic neuroblastoma. *MYCN* amplification alone, however, is insufficient for pretreatment risk stratification. Chromosome band 11q23 deletion has recently been included in the risk stratification of neuroblastoma. In the present study, we aimed to evaluate the biological characteristics and prognostic impact of 11q23 deletion and *MYCN* amplification in patients with bone marrow metastatic neuroblastoma.

**Methods:**

We analyzed the *MYCN* and 11q23 statuses of 101 patients with bone marrow metastatic neuroblastoma using interphase FISH of bone marrow cells. We specifically compared the biological characteristics and prognostic impact of both aberrations.

**Results:**

*MYCN* amplification and 11q23 deletion were seen in 12 (11.9%) and 40 (39.6%) patients. The two markers were mutually exclusive. *MYCN* amplification occurred mainly in patients with high lactate dehydrogenase (LDH) and high neuron-specific enolase (NSE) levels (both *P *< 0.001), and *MYCN*-amplified patients had more events (tumor relapse, progression, or death) than *MYCN*-normal patients (*P *= 0.004). 11q23 deletion was associated only with age (*P *= 0.001). Patients with *MYCN* amplification had poorer outcomes than those with normal *MYCN* (3-year event-free survival [EFS] rate: 8.3 ± 8.0% vs. 43.8 ± 8.5%, *P* < 0.001; 3-year overall survival [OS] rate: 10.4 ± 9.7% vs. 63.5% ± 5.7%, *P* < 0.001). 11q23 deletion reflected a poor prognosis only for patients with normal *MYCN* (3-year EFS rate: 34.3 ± 9.5% vs. 53.4 ± 10.3%, *P *= 0.037; 3-year OS rate: 42.9 ± 10.4% vs. 75.9 ± 6.1%, *P *= 0.048). Those with both *MYCN* amplification and 11q23 deletion had the worst outcome (*P *< 0.001).

**Conclusions:**

Chromosome band 11q23 deletion predicts poor prognosis only in bone marrow metastatic neuroblastoma patients without MYCN amplification. Combined assessment of the two markers was much superior to single-marker assessment in recognizing the patients at a high risk of disease progression.

## Background

Neuroblastoma is the most common extracranial malignancy in children with remarkable heterogeneity in clinical behaviors. Neuroblastoma accounts for 10%–15% of all pediatric cancer-related deaths [1, 2]. Although some of the tumors can regress spontaneously, most patients’ diseasees progress aggressively despite multimodality treatment, which may include chemotherapy, radiotherapy, surgical resection, hematopoietic stem cell transplantation, and immunotherapy [3, 4]. In the clinic, half of neuroblastoma patients present with metastasis at diagnosis, and less than 50% survive 5 years [5, 6].

Recently, many prognostic variables have been used for risk stratification to predict the survival of patients with neuroblastoma. Among them, genetic features such as the status of the v-myc avian myelocytomatosis viral oncogene neuroblastoma derived homolog (*MYCN*) gene [7], 11q23 allele status [8], and tumor ploidy [9, 10] are the most significant and clinically relevant factors. However, for pediatric patients with metastatic neuroblastoma who need chemotherapy prior to surgery, timely identification of genetic aberrations is not possible because of the difficulty in obtaining tumor biopsies. In addition, the results of genetic aberration identification may be inaccurate after the patients have received chemotherapy, leading to insufficient dosage and period of treatment [11, 12].

For more than two decades, a number of genetic aberrations closely associated with outcome have been identified in neuroblastoma; for the *MYCN* gene, these include 17q gain, loss of 3p, and 11q deletion [13]. Among them, 11q deletion is the most frequent, occurring in approximately 40% of primary neuroblastomas [14], and is associated with poor prognosis [15, 16]. However, the clinical significance of 11q deletion is unclear in neuroblastoma metastatic to bone marrow. What’s more, 11q deletion is not routinely examined when genome-wide methods are used to evaluate genetic aberrations [17]. 11q23 is the most commonly deleted 11q region, and interphase fluorescence in situ hybridization (FISH) has been previously confirmed as a reliable method for detecting *MYCN* status in bone marrow metastases of neuroblastoma [18, 19]. Therefore, in this study, we used FISH to simultaneously detect *MYCN* and 11q23 deletion in bone marrow cells in order to evaluate the biological characteristics and survival associated with these two markers.

## Materials and methods

### Patients and treatment protocols

Consecutive children with neuroblastoma newly diagnosed at the Hematology Oncology Center, Beijing Children’s Hospital, Capital Medical University between January 2015 and December 2016 were selected. All patients were diagnosed according to the International Neuroblastoma Staging System [20]. The criteria for patient inclusion were ≥ 20% neuroblastoma cells in the bone marrow samples and treatment according to the BCH-NB-2007 protocol (based on the Hong Kong neuroblastoma protocol) [21].

The BCH-NB-2007 protocol for newly diagnosed high-risk neuroblastoma was comprised of 7 cycles of intensive chemotherapy: CAV regimen [cyclophosphamide (70 mg/kg) on days 1–2, adriamycin (25 mg/m^2^) on days 1–3, and vincristine (0.033 mg/kg)] on days 1–3 for cycles 1, 2, 4, and 6; CVP regimen [cisplatin (50 mg/m^2^) on days 1–4 and VP16 (200 mg/m^2^) on days 1–3] for cycles 3, 5, and 7. Primary tumor resection was performed after 4 cycles of chemotherapy; peripheral blood stem cells (PBSCs) were then harvested for possible autologous bone marrow transplantation. Autologous PBSC transplantation (PBSCT) was performed after 7 cycles of chemotherapy. The patients received local irradiation (20–25 Gy) at the site of primary tumor as indicated [22]. At 4–5 weeks after autologous PBSCT, they received maintenance chemotherapy with cis-retinoic acid (160 mg/m^2^) for 14 days alternating with 14 days off for 9–12 months.

The treatment protocol for low-/intermediate-risk neuroblastoma was comprised of 2–3 cycles of CBVP regimen [carboplatin (200 mg/m^2^) and etoposide (150 mg/m^2^)] and CADO regimen [vincristine (1.5 mg/m^2^) and adriamycin (25 mg/m^2^)] used alternately. Primary tumor resection was usually performed after chemotherapy. If surgery was performed before chemotherapy, 2–3 cycles of chemotherapy for low-risk patients and 4–6 cycles for intermediate-risk patients were needed.

All patients were treated and evaluated according to the BCH-NB-2007 protocol. After the treatment, the patients were followed-up every 3 months mainly by outpatient follow-up visits (bone marrow examination and computed tomography or magnetic resonance imaging) and telephone interview. The final date for data collection was August 31, 2018. This study was approved by the Beijing Children’s Hospital Institutional Ethics Committee. Informed consent was obtained from the parents or their guardians in accordance with the Declaration of Helsinki.

### Bone marrow sample processing

Bone marrow samples of all patients obtained by bone marrow puncture were collected in heparin-coated tubes at diagnosis. The sample was immediately shaken gently after collection to avoid bone marrow agglutination. Then, the cells were cultured in 8 mL RPMI-1640 (Hyclone, Beijing, China) and 2 mL fetal bovine serum medium (Invitrogen, Carlsbad, CA, USA) for 24 h. The cell density was (1 − 3) × 10^6^/mL, and the total amount of cells was about (1 − 3) × 10^7^. Colchicine was added at 2 h before termination of the culture. The cells were then treated with 0.075 mol/L KCl for 30 min and fixed twice in a 3:1 mixture of methanol:acetic acid. Finally, the cell suspension was stored in a fresh fixative solution at 4 °C. Bone marrow biopsies were prepared on wax blocks for morphological examination.

### FISH analysis of bone marrow cells

The FISH technique was performed as previously reported [17]. The statuses of *MYCN* and 11q were determined with DNA probes from Vysis [N-MYC(2p24)/CEP2(2p11.1-q11.1) Dual Color Probe and LSI MLL Dual Color, Break Apart Rearrangement Probe] (cat. No. 7J72-01 and 8L57-20, Abbott Laboratories, Abbott Park, IL, USA). A Leica DM6000B microscope (Leica Microsystems GmbH, Wetzlar, Germany) was used to take fluorescence images. According to the recommendations of the European Neuroblastoma Quality Assessment group [23, 24], *MYCN* amplification was defined as a > fourfold increase of *MYCN* signals in relation to the number of chromosome 2, and 11q23 deletion was defined as only one fusion signal displayed.

### Karyotype analysis of bone marrow cells

Chromosome karyotype analysis of bone marrow cells was performed on G-banded preparations. At least 20 metaphases were analyzed in detail. The karyotype results were interpreted according to the International System for Human Cytogenomic Nomenclature guidelines (2016) [25]. The clones containing at least 2 cells with the same additional chromosome or structural abnormality or at least 3 cells with the same missing chromosome were defined as abnormal clones.

### Morphologic analysis and diagnostic biomarker detection

Microscopic examination of bone marrow biopsies to determine the presence of neuroblastoma cells was done by at least 2 independent laboratory experts. Serum tumor markers such as lactate dehydrogenase (LDH) and neuron specific enolase (NSE) levels were detected by radioimmunoassay and full-automatic biochemical analysis at the time of diagnosis in all patients.

### Statistical analysis

All statistical analyses were performed using the SPSS software, version 16.0 (SPSS Inc., Chicago, IL, USA). Comparisons of patients with or without 11q23 deletion and *MYCN* amplification and the association of clinical characteristics were evaluated using the χ^2^ test. Event-free survival (EFS) was defined from the date of diagnosis to the date of one of the following events: progression, relapse, death, or last contact with patients in continuous complete remission (CR). Overall survival (OS) was defined from the date of diagnosis to the date of death due to any reason or the last contact with patients in continuous CR. Kaplan–Meier survival analysis and log-rank test were used to determine the differences in EFS and OS in all patients by MYCN and 11q23 statuses. Multivariate backward Cox regression analysis model was built which included only those variables reaching a *P* value < 0.05 in the respective univariate analysis. All tests with a *P *< 0.05 in two-sided distributions were considered statistically significant.

## Results

### Clinical characteristics of 101 patients with bone marrow metastatic neuroblastoma

Among the 161 patients with newly diagnosed neuroblastoma, 45 had no bone marrow metastasis, 13 gave up treatment after diagnosis, and 2 were lost to follow-up. A total of 101 patients with stage IV neuroblastoma were thereby analyzed, including 57 males and 44 females in a ratio of 1.3:1. The patients were aged 7–105 months (median, 36 months), and 85 (84.2%) were older than 18 months. The median white blood cell (WBC) count was 5.6 × 10^9^/L (range 1.7–42.5 × 10^9^/L) with 60 (59.4%) patients having normal WBC. Only 8 (7.9%) patients had hemoglobin > 120 g/L. The rest had different degrees of anemia, but most cases were mild. The median hemoglobin level was 94 g/L (range 58–135 g/L). The amount of platelets ranged from 37 × 10^9^/L to 622 × 10^9^/L (median, 275 × 10^9^/L). Almost all patients (92.1%) were grouped as having high-risk neuroblastoma. The primary tumor sites were the abdomen for 88 patients, thorax for 12 patients, and neck for 1 patient. 11q23 deletion was seen in 40 (39.6%) patients, and *MYCN* amplification in 12 (11.9%). In terms of clinical serum tumor markers, only 22 (21.8%) patients had LDH levels ≥ 1500 IU/L, and 41 (40.6%) had NSE levels **≥** 370 ng/mL.

Each patient received a total of 7 cycles of chemotherapy except for those who died during therapy. During follow-up, 39 (38.6%) patients died, and 13 (12.9%) suffered from tumor relapse or progression; the other 49 (48.5%) had stable disease. Among the patients who died, 11 died of tumor progression during intensive chemotherapy, 24 died of tumor progression during maintenance treatment, and 4 died of tumor recurrence after the completion of chemotherapy. Of note, 4 of the 24 patients who died during maintenance treatment had intracranial metastasis. The main clinical characteristics of the 101 patients are shown in Table 1.

**Table 1:** Baseline characteristics of the 101 patients with bone marrow metastatic neuroblastoma

Characteristic	Total [cases (%)]
Age (months)	
< 18	16 (15.8)
≥ 18	85 (84.2)
Sex	
Male	57 (56.4)
Female	44 (43.6)
WBC (× 10^9^/L)	
> 10	17 (16.8)
4–10	60 (59.4)
< 4	24 (23.8)
Hemoglobin (g/L)	
> 120	8 (7.9)
90–120	59 (58.4)
60–89	33 (32.7)
< 60	1 (1.0)
Platelet (× 10^9^/L)	
> 300	47 (46.5)
100–300	48 (47.5)
< 100	6 (6.0)
Risk stratification	
Low-risk	1 (1.0)
Intermediate-risk	7 (6.9)
High-risk	93 (92.1)
Primary tumor site	
Abdomen	88 (87.1)
Thorax	12 (11.9)
Neck	1 (1.0)
11q23 deletion	
Yes	40 (39.6)
No	61 (60.4)
*MYCN* amplification	
Yes	12 (11.9)
No	89 (88.1)
LDH (IU/L)	
< 1500	79 (78.2)
≥ 1500	22 (21.8)
NSE (ng/mL)	
< 370	60 (59.4)
≥ 370	41 (40.6)
Event	
No event	49 (48.5)
Relapse/progression	13 (12.9)
Death	39 (38.6)

*WBC* white blood cell, *MYCN* the v-myc avian myelocytomatosis viral oncogene neuroblastoma derived homolog, *LDH* lactate dehydrogenase, *NSE* neuron-specific enolase

### Genetic markers of *MYCN* and 11q23 in bone marrow cells

**Fig. 1:**
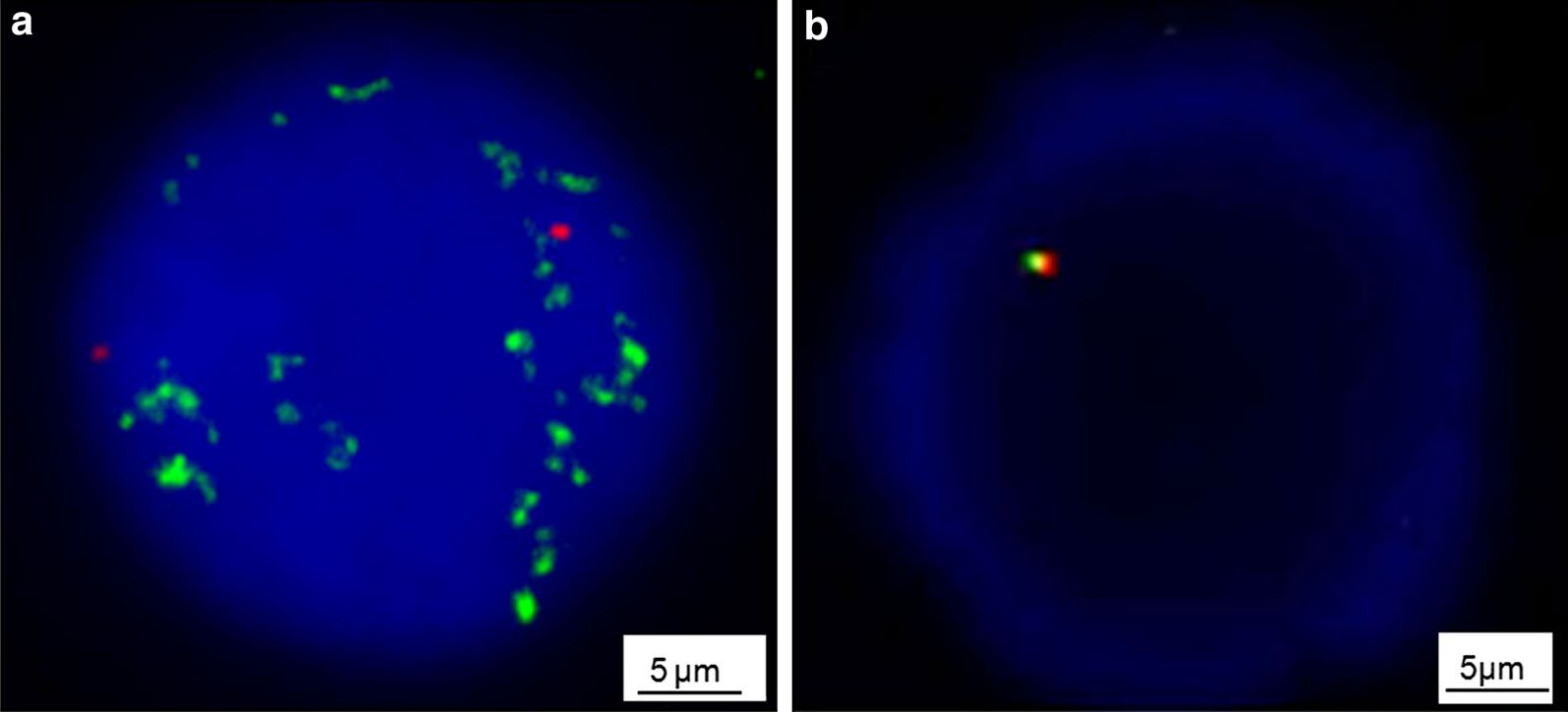
Typical fluorescence in situ hybridization (FISH) images of bone marrow cells from patients with bone marrow metastatic neuroblastoma. **a** The status of v-myc avian myelocytomatosis viral oncogene neuroblastoma derived homolog (*MYCN*) amplification was detected using a dual-color probe. Green signals represent the specific probe for *MYCN*, and red signals stand for centromeric chromosome 2 probes. *MYCN* signals show more than 10 copies within the nuclei. **b** The status of 11q23 deletion was also detected using a dual-color probe. Normal cells show a two orange/green fusion signal pattern. In a cell with 11q23 deletion, the pattern is one orange/green fusion signal

In all patients, interphase FISH detected the *MYCN* gene and 11q23 status of bone marrow cells. Chromosome karyotype analysis was performed in 55 patients. *MYCN* amplification was seen in 12 (11.9%) patients, with > 30 gene copies/cell (Fig. 1a). *MYCN* gain was seen in 21 (20.8%) patients, with 2–9 signal copies. Among the 101 patients, 40 (39.6%) had 11q23 deletion (Fig. 1b). Only 2 (2.0%) patients had both *MYCN* amplification and 11q23 deletion. *MYCN* amplification and 11q23 deletion were not associated with higher percentages of tumor cells in bone marrow or more severe hematopoietic suppression (both *P* > 0.05).

### Relationship of *MYCN* and 11q23 statuses with clinicobiological characteristics of patients with bone marrow metastatic neuroblastoma

We observed no association of *MYCN* amplification with age, sex, or primary tumor site (Table 2). However, the primary tumor site of all patients with *MYCN* amplification was in the abdomen. We found that *MYCN* amplification mainly occurred in those with high LDH and NSE levels (both *P *< 0.001), and more events were observed in patients with *MYCN* amplification than in those with normal *MYCN* (*P *= 0.004).

**Table 2:** Clinical characteristics of bone marrow metastatic neuroblastoma patients according to *MYCN* and 11q23 statuses

Variable	*MYCN* status [cases (%)]		*P* value*	11q23 status [cases (%)]		*P* value*
	Amplified	Normal		Deleted	Normal	
Total	12	89		40	61	
Sex						
Male	7 (58.3)	50 (56.2)	0.536	24 (60.0)	33 (54.1)	0.682
Female	5 (41.7)	39 (43.8)		16 (40.0)	28 (45.9)	
Age (months)						
< 18	4 (33.3)	12 (13.5)	0.095	2 (5.0)	14 (23.0)	0.024
≥ 18	8 (66.7)	77 (86.5)		38 (95.0)	47 (77.0)	
Primary tumor site						
Abdomen	12 (100.0)	76 (85.4)	0.436	33 (82.5)	55 (90.2)	0.339
Thorax	0 (0)	12 (13.5)		6 (15.0)	6 (9.8)	
Neck	0 (0)	1 (1.1)		1 (2.5)	0 (0)	
LDH (IU/L)						
< 1500	1 (8.3)	78 (87.6)	<0.001	32 (80.0)	47 (77.0)	0.808
≥ 1500	11 (91.7)	11 (12.4)		8 (20.0)	14 (23.0)	
NSE (ng/mL)						
< 370	1 (8.3)	59 (66.3)	< 0.001	21 (52.5)	39 (63.9)	0.302
≥ 370	11 (91.7)	30 (33.7)		19 (47.5)	22 (36.1)	
Event						
No	1 (8.3)	48 (53.9)	0.004	16 (40.0)	33 (54.1)	0.118
Yes	11 (91.7)	41 (46.1)		24 (60.0)	28 (45.9)	

*MYCN* the v-myc avian myelocytomatosis viral oncogene neuroblastoma derived homolog, *LDH* lactate dehydrogenase, *NSE* neuron-specific enolase

* The χ^2^ test was used for statistical analysis

Unlike the *MYCN* gene, 11q23 deletion was associated with age (*P *= 0.024). We found 38 (95.0%) cases of 11q23 deletion occurred in children older than 18 months. The median age at diagnosis was 41 months for patients with 11q23 deletion, but 24.5 months for those with *MYCN* amplification. Although 24 (60.0%) patients with 11q23 deletion had events occurred, no significant difference was observed between patients with 11q23 deletion and normal 11q23 (*P* = 0.118).

### Impact of *MYCN* and 11q23 on prognosis of bone marrow metastatic neuroblastoma

**Fig. 2:**
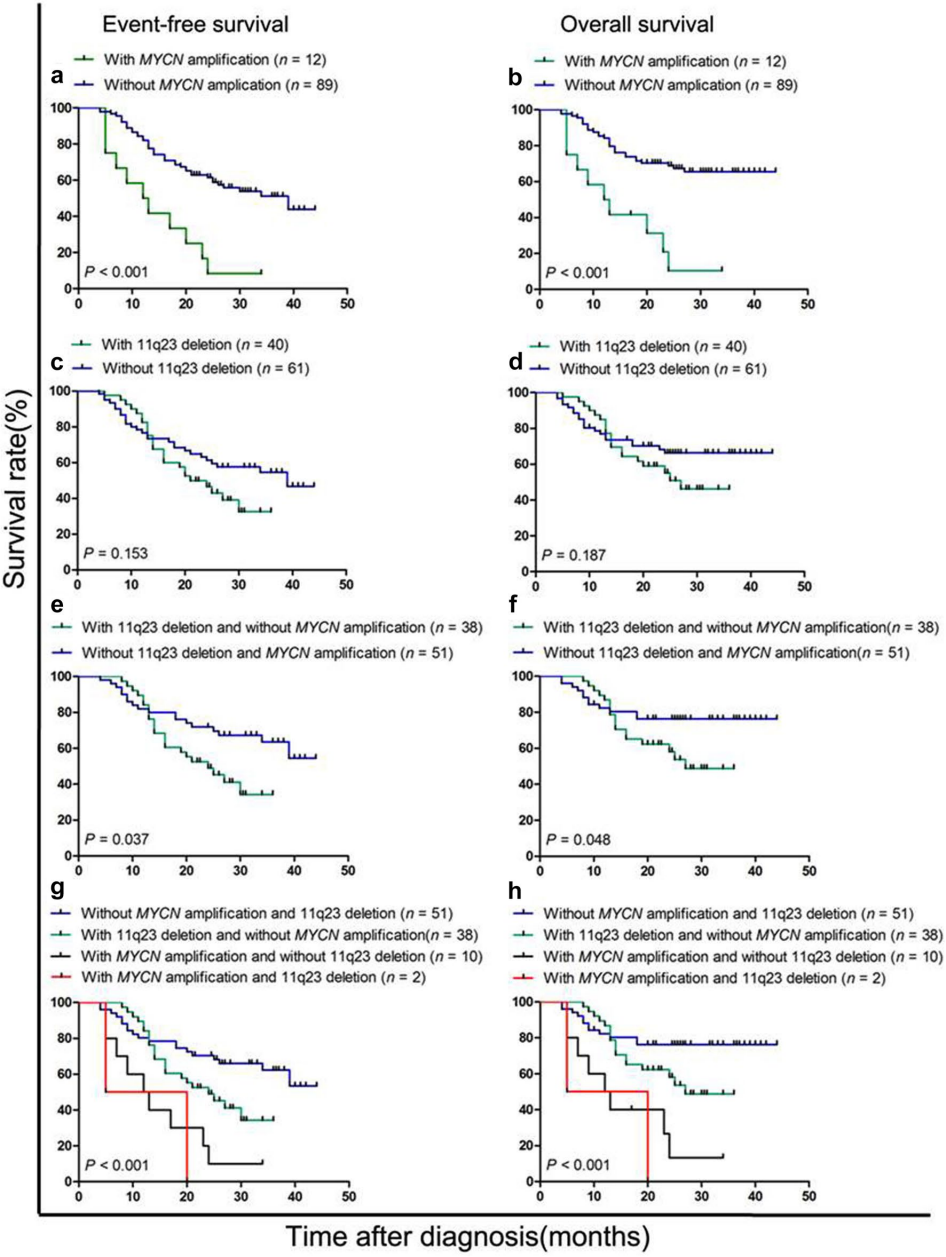
The prognostic significance of *MYCN* amplification and 11q23 deletion in patients with bone marrow metastatic neuroblastoma. **a**, **b** Event-free survival (EFS) and overall survival (OS) of the 101 patients stratified by *MYCN* gene. **c**, **d** EFS and OS of the 101 patients stratified by 11q23 status. **e**, **f** EFS and OS of the 89 patients without MYCN amplification stratified by 11q23 status. **g**, **h** EFS and OS of the 101 patients stratified by combined assessment of *MYCN* and 11q23 statuses

The 3-year EFS and OS rates of the 101 patients were 45.9 ± 5.5% and 59.1 ± 5.2%, with a median follow-up time of 23 months (range, 4–44 months). The estimated 3-year EFS and OS rates of patients with *MYCN* amplification were obviously lower than those of patients with normal *MYCN* (8.3 ± 8.0% vs. 43.8 ± 8.5% and 10.4 ± 9.7% vs. 63.5% ± 5.7%, both *P* < 0.001) (Fig. 2a, b). The difference in survival was not significant between patients with and without 11q23 deletion (Fig. 2c, d). The median EFS of patients with and without *MYCN* amplification was 12.5 and 25 months, respectively. The median EFS of patients with and without 11q23 deletion was 20.5 and 26 months, respectively.

To better understand the effect of 11q23 deletion on prognosis of bone marrow metastatic neuroblastoma, we performed further survival analysis of the 89 patients without *MYCN* amplification. We surprisingly found that 11q23 deletion was a powerful prognostic factor if the influence of the *MYCN* gene was excluded. The estimated 3-year EFS and OS rates of the 38 patients with 11q23 deletion were significantly lower than those of the 51 patients without 11q23 deletion (34.3 ± 9.5% vs. 53.4 ± 10.3%, *P *= 0.037; 42.9 ± 10.4% vs. 75.9 ± 6.1%, *P *= 0.048) (Fig. 2e, f).

We then investigated the prognostic value of the combination of *MYCN* and 11q23. The 101 patients were stratified into four groups according to the statuses of *MYCN* and 11q23. The four groups differed significantly in survival (both *P *< 0.001) (Fig. 2g, h). 51 patients without *MYCN* amplification and 11q23 deletion had the best prognosis (3-year EFS and OS rates were 53.4 ± 10.3% and 73.0 ± 6.2%). The 2 patients with both *MYCN* amplification and 11q23 deletion had the worst outcome (3-year EFS and OS rates were both 0). Multivariate analysis showed that only *MYCN* amplification was an independent prognostic factor of EFS for all patients (Table 3). 11q23 deletion was a prognostic factor in univariate analysis only for patients without *MYCN* amplification, but it was not an independent prognostic factor in the multivariate analysis.

**Table 3:** Univariate and multivariate analysis of event-free survival (EFS) of children with bone marrow metastatic neuroblastoma

Variable	Total patients (*n* = 101)				Patients without *MYCN* amplification (*n* = 89)			
	Univariate*	Multivariate^#^			Univariate*	Multivariate^#^		
	*P*	HR	95% CI	*P*	*P*	HR	95% CI	*P*
MYCN status (amplification vs. normal)	< 0.001	2.399	1.113–5.169	0.032	–	–	–	–
LDH (≥ 1500 vs. < 1500 IU/L)	< 0.001	1.543	0.622–3.83	0.351	0.040	1.488	0.564–3.926	0.422
NSE (≥ 370 vs. < 370 ng/mL)	0.001	1.829	0.972–3.44	0.068	0.025	2.016	1.074–3.784	0.033
11q23 status (deletion vs. normal)	0.153	–	–	–	0.037	1.674	0.869–3.225	0.124

*MYCN* the v-myc avian myelocytomatosis viral oncogene neuroblastoma derived homolog; LDH, lactate dehydrogenase, *NSE* neuron-specific enolase, *HR* hazard ratio, *CI* confidence interval

* Univariate analysis for EFS was performed using the Kaplan–Meier log-rank test. All factors with *P* < 0.05 in the univariate analysis are shown

^#^All factors with *P *< 0.05 in the univariate analysis were selected in the backward Cox regression model for multivariate analysis of EFS

## Discussion

In recent years, the pretreatment risk stratification for neuroblastoma patients has become increasingly necessary [26, 27]. Currently, the identified biological factors closely related to prognosis include age, histology, ploidy, *MYCN* gene and specific segmental chromosomal aberrations [28, 29]. In the present study, we evaluated the prognostic value of 11q23 deletion with or without MYCN amplification in bone marrow metastatic neuroblastoma. We found that 11q23 deletion was a prognostic marker only for patients without *MYCN* amplification and that 11q23 deletion was closely associated with age. Almost all cases of 11q23 deletion occurred in children older than 18 months. Multivariate analyses for EFS indicated that *MYCN* amplification was an independent prognostic factor, but 11q23 deletion was not. Combined assessment of *MYCN* and 11q23 deletion was much better than single-marker assessment in identifying the patients at a high risk of disease progression in bone marrow metastatic neuroblastoma.

In the present study, *MYCN* amplification was present in 11.9% of the patients, which was lower than those in our previous study [17] and other reports [21, 30]. 11q23 deletion was found in 39.6% of the patients, a rate close to those reported by other groups [11, 31]. Similar to another group [31], we here found that 11q23 deletion occurred commonly in patients with normal *MYCN*. In fact, the two markers were mutually exclusive; only 2 patients presented with both. We thought that *MYCN* amplification and 11q23 deletion were distinct genetic aberrations with different clinicobiological characteristics. *MYCN* amplification was associated with LDH and NSE levels, whereas 11q23 deletion was associated with age. There was a marked difference in age at diagnosis between the two groups, with a median age of 24.5 months in the *MYCN* amplification group and 41 months in the 11q23 deletion group.

We further confirmed that 11q23 deletion constituted a distinct group of patients with unfavorable neuroblastoma. In the present study, *MYCN* amplification was confirmed as an independent prognostic factor. We found no obvious differences in EFS and OS between the 11q23 deletion and the 11q23 normal groups. However, we found that 11q23 deletion was a powerful marker for poor prognosis in patients without *MYCN* amplification. Among the 89 patients without *MYCN* amplification, the 3-year EFS and OS rates were significantly lower in patients with 11q23 deletion than in those without the deletion (both *P *< 0.05).

It was reported that 11q23 deletion may be involved in the dysregulation of important genes, including tumor suppressor genes and oncogenes [32]. One candidate gene is forkhead box R1 (*FOXR1*), which is located in 11q23.3. 11q23.3 deletion results in either mixed-lineage leukemia (*MLL*) or platelet activating factor acetylhydrolase 1b catalytic subunit 2 (*PAFAH1B2*) fusing to the proximal side of *FOXR1*. The new fusion transcripts stimulate the overexpression of *FOXR1*. Functional analyses have shown that *FOXR1* overexpression could promote cell proliferation and may be a tumor-driving event [33]. Another important tumor suppressor gene is h2a histone family member X (*H2AFX*), which resides in the 11q23.2-q23.3 region. *H2AFX* participates in DNA double-strand breaks (DSBs) through phosphorylation. The loss of *H2AFX* promotes tumorigenesis due to defective DSB repair, increased radiation sensitivity, and genomic instability [10]. It was reported that more chromosomal breaks were seen in neuroblastoma patients with 11q23 deletion, which may be explained by genomic instability related to the loss of *H2AFX* gene [34]. This hypothesis also implied that 11q23 deletion occurred early in tumorigenesis. However, in the present study, the median age at diagnosis was 41 months for patients with 11q23 deletion and 24.5 months for those with *MYCN* amplification. The definitive role of 11q23 deletion in tumorigenesis of neuroblastoma still needs to be elucidated.

The present study showed that a large number of patients with *MYCN* amplification or 11q23 deletion had unfavorable prognosis. The survival rate was higher in patients with 11q23 deletion than in those with *MYCN* amplification. However, OS has been reported to be similar for patients with the two aberrations after 8 years of follow-up [10]. We further evaluated the prognostic value of combined assessment of *MYCN* and 11q23 statuses and found that the combined assessment was much superior to single-marker assessment in recognizing the patients at a high risk of disease progression.

The present study investigated the outcomes of bone marrow metastatic neuroblastoma patients with 11q23 deletion and of the rare patients with both *MYCN* amplification and 11q23 deletion. However, there were some limitations of this study. First, it is unclear how the results would be used to affect treatment. Second, the cases of both MYCN amplification and 11q23 deletion were limited. In our future study, we will focus on how further subdivision of these patients would alter treatment.

## Conclusions

In the present study, we found that for patients with bone marrow metastatic neuroblastoma, 11q23 deletion predicted poor prognosis only in those without *MYCN* amplification. Combined assessment of the two markers was superior to single-marker assessment in identifying patients at a high risk of disease progression.

## Data Availability

The datasets used and/or analyzed during the current study are available from the corresponding author on reasonable request.

## Abbreviations

MYCN: V-Myc avian myelocytomatosis viral oncogene neuroblastoma derived homolog
FISH: fluorescence in situ hybridization
HR: high-risk
PBSC: peripheral blood stem cells
PBSCT: peripheral blood stem cell transplantation
LDH: lactate dehydrogenase
NSE: neuron specific enolase
EFS: event-free survival
CR: complete remission
OS: overall survival
WBC: white blood cell
